# Use of albumin in spontaneous bacterial peritonitis is cost-effective

**DOI:** 10.1186/cc14433

**Published:** 2015-03-16

**Authors:** A Farrugia, M Bansal, P Caraceni

**Affiliations:** 1 University of Western Australia, Perth, Australia; 2 Thought Semantics LLC, Sterling, VA, USA; 3 University of Bologna, Italy

## Introduction

Assessing the cost-effectiveness of therapeutic interventions is increasingly crucial for health decision-making. Spontaneous bacterial peritonitis (SBP) is one of the major complications of liver cirrhosis. The use of albumin in conjunction with antibiotics has been shown to be effective through clinical trials [1].

## Methods

**Figure 1:**
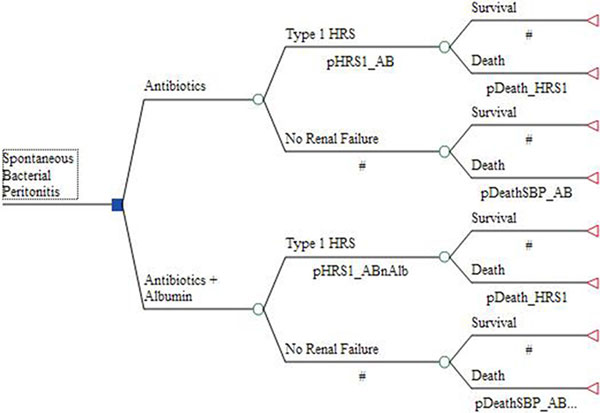
**Structure of decision tree for patients with SBP**.

A decision tree (TreeAge®) (Figure 1) was populated from published sources for clinical, cost and epidemiologic variables. The perspective taken was that of the US payer. The robustness of the model was checked using one-way and probabilistic sensitivity analyses. The clinical course was followed for 3 months or until death. Total medical costs and quality-adjusted life years (QALYs) [2] were calculated.

## Results

**Figure 2:**
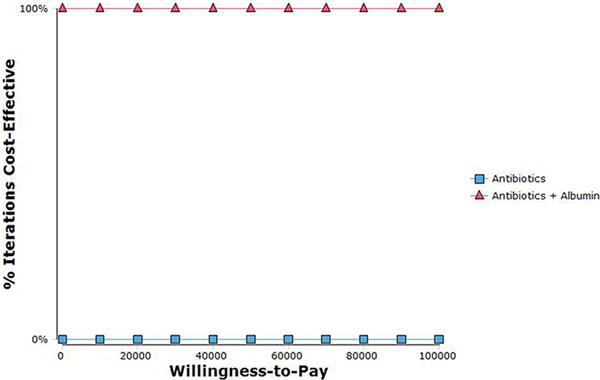
**Cost-effectiveness acceptability across range of WTP**.

Total costs were decreased when using albumin, and the improved survival resulted in an additional QALY for patients on albumin, decreasing the cost per QALY. See Table 1 and Figure 2.

**Table 1:** results of the cost-effectiveness model.

Treatment	QALYs	Total medical costs ($)	Total costs/ QALY ($)
Antibiotics + albumin	2.45	7,628	3,111
Antibiotics	1.48	7,682	5,182

## Conclusion

The use of albumin in the treatment of SPB is cost-effective.

## References

[B1] Poca M Clin Gastroenterol Hepatol 2012 10 309 15 10.1016/j.cgh.2011.11.012 22094025

[B2] Wells CD Dig Dis Sci 2004 49 453 8 15139497 10.1023/b:ddas.0000020502.46886.c1

